# Baseline study of morphometric traits of wild *Capsicum annuum* growing near two biosphere reserves in the Peninsula of Baja California for future conservation management

**DOI:** 10.1186/s12870-015-0505-6

**Published:** 2015-05-10

**Authors:** Bernardo Murillo-Amador, Edgar Omar Rueda-Puente, Enrique Troyo-Diéguez, Miguel Víctor Córdoba-Matson, Luis Guillermo Hernández-Montiel, Alejandra Nieto-Garibay

**Affiliations:** Centro de Investigaciones Biológicas del Noroeste, S.C. La Paz, La Paz, Baja California Sur México; Universidad de Sonora, Hermosillo, Sonora México

**Keywords:** Solanaceae, Mineral content, Growth type, Vegetation associated

## Abstract

**Background:**

Despite the ecological and socioeconomic importance of wild *Capsicum annuum* L., few investigations have been carried out to study basic characteristics. The peninsula of Baja California has a unique characteristic that it provides a high degree of isolation for the development of unique highly diverse endemic populations. The objective of this study was to evaluate for the first time the growth type, associated vegetation, morphometric traits in plants, in fruits and mineral content of roots, stems and leaves of three wild populations of *Capsicum* in Baja California, Mexico, near biosphere reserves.

**Results:**

The results showed that the majority of plants of wild *Capsicum annuum* have a shrub growth type and were associated with communities consisting of 43 species of 20 families the most representative being Fabaceae, Cactaceae and Euphorbiaceae. Significant differences between populations were found in plant height, main stem diameter, beginning of canopy, leaf area, leaf average and maximum width, stems and roots dry weights. Coverage, leaf length and dry weight did not show differences. Potassium, sodium and zinc showed significant differences between populations in their roots, stems and leaves, while magnesium and manganese showed significant differences only in roots and stems, iron in stems and leaves, calcium in roots and leaves and phosphorus did not show differences. Average fruit weight, length, 100 fruits dry weight, 100 fruits pulp dry weight and pulp/seeds ratio showed significant differences between populations, while fruit number, average fruit fresh weight, peduncle length, fruit width, seeds per fruit and seed dry weight, did not show differences.

**Conclusions:**

We concluded that this study of traits of wild *Capsicum*, provides useful information of morphometric variation between wild populations that will be of value for future decision processes involved in the management and preservation of germplasm and genetic resources.

## Background

The genus Capsicum (Solanaceae) contains a large number of cultivated species as well as wild species that are grown for their fruits, and are an important vegetable consumed throughout the world. There are about 30 species of *Capsicum*, but only *C. annuum*, *C. frutescens*, *C. chinense* Jacq., *C. baccatum*, and *C. pubescens* Ruiz et Pav are presently domesticated.

*Capsicum annuum* has the highest morphometric diversity and is widely cultivated in America, Asia, Africa, and Mediterranean countries for their fruits that have numerous uses in culinary preparations. It is a good source of starch, dietary fiber, protein, lipids, and minerals. In addition to their nutritive value, they contain phytochemicals with antioxidant properties that are beneficial to human health [1].

In general, wild *Capsicum* species are found at low altitudes, rarely exceeding 1000 m.a.s.l. [2,3]. Botanically, *C. annuum* species are tender perennials when grown in their native tropical habitats but are also commonly grown as annual crops in parts of the world where frost and freezing temperatures preclude year-round field production [4], they range extends from USA to Peru.

In México, *Capsicum* peppers are cultivated and can be found in the wild. Wild populations of *C. annuum* are widely distributed in Mexico, growing in dry tropical forests, in desert scrubs, near roads, home gardens, pasturelands, and around crop fields [5]. They produce small round berries held erect on long pedicels, that are deciduous, brilliant red when ripe. They are extremely hot to the taste, and they stand out of the foliage allowing for easy harvesting during ripeness [2,6,7]. They are very attractive for birds and are consumed by frugivorous birds species, which are the main seed dispersers [7-9]. Therefore it is necessary to harvest berries before they mature. Moreover the berries tend to fall from the plant when they mature [10].

In northeastern Mexico wild *Capsicum* species are important resources for people living in rural communities because there is little farm work and employment is scarce [11]. Fruits of this species are consumed fresh,dried or processed in vinegar or sauce representing a promising potential market both in Mexico and USA [12,13]. In Baja California Sur, México, wild *C. annuum* is called “chilpitín” or “chiltepín”. and represent a wild chili that come from small shrubs with highly branched stems, with alternate petiole leaves. Flowering occurs almost year round, with white flowers and five lobes. The fruit grows in streams and is distributed in tropical areas of the Cape Region of the Baja California Peninsula [14] and is well accepted for different culinary [14] and medicinal [15] purposes. According to the Missouri Botanical Garden, the wild *Capsicum* species found in Baja California Sur, México is *C. annuum* var. *aviculare* (Dierb.) D’Arcy & Eshbaugh, native from Mesoamerica with a distribution range extending from the south of the United States to the north of South America [7,16]. However, Kraft *et al*. [17] reported that some accessions were a different phenotype although collected in Baja California Sur. Generally speaking, these accessions collected were morphometrically similar (with similar cultural use, but not commercialized in any significant manner) to those found in Sonora and Arizona (*C. annuum* var. *glabriusculum*). However, according to the Missouri Botanical Garden, *aviculare* and *glabrisuculum* are accepted synonyms.

Chiltepín production in Mexico has been estimated to be 50 t yr^−1^, it is an important crop product for subsistence farmers of the central and northern regions of the country [7,18-20]. The agronomic interest of chiltepín exceeds its value as a local commodity, as it is genetically compatible with the domesticated varieties of *C. annuum*. Wild *Capsicum* species are important reservoirs of genes and sources of genetic diversity for breeding programs of cultivated pepper, as sources of resistance against pests, pathogens [21,22], adverse environmental factors, and for increasing quality and quantity of production [23,24]. Maiti *et al*. [25] stated that piquín pepper might be considered as a new crop because it has been exploited for many years in its wild form. Extensive commercial farming of piquín pepper does not exist. Almost all piquín production comes from harvesting of wild plants, usually with overexploitation conditions, causing loss of biodiversity [11].

The current main limitation for planting piquín as a commercial crop is its low seed germination (dormancy). In addition, research on developing production technology for piquín is limited. Although a perennial plant it can die in times of drought or even in the winter. It sprouts with the first rains and full production occurs at the end of the rainy season from August to December, depending of locality. When it is fresh it is of green color and when dry color changes to red. The piquín pepper is found in the local markets at the end of the season of rains [26]. Domestication causes dispersal from center of origin [27,28] causing artificial selection that has led to changes in their mating systems, dispersal mechanisms, physiology, and their genetic structure [23,29].

For this reason, it is important to know the extent and distribution of genetic variation among populations since it is crucial for understanding the origin and evolution of plant populations in natural conditions. The information about where it grows, its commercial variants and their wild relatives is important for potential breeders, population geneticists, and conservation biologists concerned with the use, management and conservation of plant genetic resources [30].

Based on the aforementioned lack of biological information such as knowledge of morphometric traits, and the relatively little research available, the objective of this study was to analyze three populations of wild *C. annuum* growing near two biosphere reserves in Baja California Sur, Mexico. The purpose of which is to generate fundamental baseline data of the chili chiltepín useful for providing a framework for germplasm use for crop management domestication and species conservation. Four specific questions were addressed: (1) what is the growth type of wild *Capsicum* plants in each population? (2) which wild species and families are more associated with wild *Capsicum* plants? (3) are there differences between mineral content and morphometric traits in plants and fruits between populations? and (4) how some environment conditions affect the growth of wild *Capsicum* plants? Undoubtedly, the results of the present study will be valuable in providing a better understanding of some of the wild *C. annuum* populations growing near two biosphere reserves in Baja California Sur, Mexico.

## Results

The MANOVA analysis for variables measured in plants (*in-situ*) showed significant differences between sample populations (Wilks = 0.155, F = 3.45, *p* = 0.01). This analysis included the variables plant height, plant coverage, main stem diameter and height of the beginning of canopy. The MANOVA analysis for morphometric traits from plants measured in laboratory such as leaf area, leaf length, average and maximum width of leaf, leaves, roots and stems dry weights showed significant differences between sample populations (Wilks = 0.036, F = 3.64, *p* = 0.01). The MANOVA analysis for those variables of fruits measured from collected plants (number of fruits per plant, average fresh and dry of fruits and peduncle length) showed significant differences between sample populations (Wilks = 0.062, F = 4.52, *p* = 0.009). The MANOVA analysis for the variables, fruit length and width, seeds per fruit, dry weight of 100 fruits, dry weight of seeds and pulp of 100 fruits, dry weight of 1000 seeds and index of pulp/seeds, measured in 400 fruits collected per population, showed significant differences between sample populations (Wilks = 0.00019, F = 30.00, *p* = 0.0002). The MANOVA analysis for mineral content in roots, stems and leaves (Ca, Mg, K, Na, Fe, Mn, Cu, Zn and P) showed significant differences between populations for roots (Wilks = 0.013, F = 3.37, *p* = 0.04), stems (Wilks = 0.022, F = 2.54, *p* = 0.05) and leaves (Wilks = 0.00078, F = 15.43, *p* = 0.00024). According with MANOVA analysis, it can be seen that the relationship of Wilks possibilities is significant at the level of *p* ≤ 0.01 or *p* ≤ 0.05.

### Vegetation associated to wild Capsicum

The results from the first study estimation indicate that *Capsicum* in the sample populations is associated with twenty wild vegetal families where Fabaceae (21.4%), Cactaceae (16.1%) and Euphorbiaceae (12.5%) are the most representative (Table 1). The results showed 43 species associated to *Capsicum* ecotypes in the populations, these being *Jatropha cinerea* (5%) the most abundant, followed by *Prosopis glandulosa* var. *torreyana*, *Erythrina flabelliformis*, *Mimosa dystachia*, *Stenocereus thurberii*, *Tecoma stands*, *Pachycereus pecten-aboriginum*, *Ambrosia ambrosioides*, *Opuntia tapona*, *Celtis reticulata*, *Bignonia unguis-cati* and *Schaeferia shrevei* (all species with 4%) the most representatives. The rest of species showed 2% of presence (Table 1). The analysis of vegetation among collection sites showed some differences in the predominant vegetation on each site, i.e. in Los Gatos, the three species most abundant from most to least were *Jatropha cinerea* > *Prosopis glandulosa* var. *torreyana* > *Pachycereus pringleii*. In San Bartolo, the predominant species were *Prosopis glandulosa* var. *torreyana* > *Pachycereus pecten-aboriginum* > *Jatropha cinerea*, while in Santiago, the three most abundant species were in the following order *Celtis reticulata* > *Tecoma stands* > *Pachycereus pecten-aboriginum*.

**Table 1:** **Main species of vegetation associated to wild** ***Capsicum*** **chili ecotypes collected in three populations near two biosphere reserves in Mexico**

**Population**	**Common name**	**Scientific name**	**Life form**	**Family**
Los Gatos	Lomboy blanco	*Jatropha cinerea*	Bush	Euphorbiaceae
Los Gatos	Mezquite	*Prosopis glandulosa* var. *torreyana*	Tree	Fabaceae
Los Gatos	Colorín or chilicote	*Erythrina flabelliformis*	Tree	Fabaceae
Los Gatos	Huerivo	*Populus brandegeei*	Tree	Salicaceae
Los Gatos	Cardón	*Pachycereus pringleii*	Cactus tree	Cactaceae
Los Gatos	Uña de gato	*Mimosa dystachia*	Tree	Fabaceae
Los Gatos	Pitahaya dulce	*Stenocereus thurberii*	Cactus tree	Cactaceae
Los Gatos	Palo adan	*Fouquieria diguetti*	Bush	Fouquieriaceae
San Bartolo	Mezquite	*Prosopis glandulosa* var. *torreyana*	Tree	Fabaceae
San Bartolo	Palo de arco	*Tecoma stands*	Tree	Bignoniaceae
San Bartolo	Cardón barbón	*Pachycereus pecten-aboriginum*	Cactus tree	Cactaceae
San Bartolo	Chicura	*Ambrosia ambrosioides*	Bush	Compositae
San Bartolo	Lomboy blanco	*Jatropha cinerea*	Bush	Euphorbiaceae
San Bartolo	Pitahaya dulce	*Stenocereus thurberii*	Cactus tree	Cactaceae
San Bartolo	Uña de gato	*Mimosa dystachia*	Tree	Fabaceae
San Bartolo	Nopal	*Opuntia tapona*	Cactus bush	Cactaceae
San Bartolo	Vainoro	*Celtis reticulata*	Tree	Ulmaceae
San Bartolo	Huirote de corral	*Bignonia unguis-cati*	Vine, annual herb	Bignoniaceae
San Bartolo	Hierba del cuervo	*Schaeferia shrevei*	Tree	Celastraceae
San Bartolo	Lentejilla	*Senna villosa*	Bush	Fabaceae
San Bartolo	Palo zorrillo	*Cassia emarginata*	Tree	Fabaceae
San Bartolo	Bernardia	*Bernardia mexicana*	Bush	Euphorbiaceae
San Bartolo	Alcager	*Pereskiopsis porterii*	Bush, cactus scandent	Cactaceae
San Bartolo	Ventamanta	*Coursetia caribaea*	Perennial herb	Fabaceae
San Bartolo	Cardoncillo	*Elytraria imbricata*	Annual herb	Acanthaceae
San Bartolo	Abutilón	*Abutilon palmeri*	Annual herb	Malvaceae
San Bartolo	Brikelia	*Brickelia coulteri*	Bush	Asteraceae
San Bartolo	Naranjillo	*Zantoxylon sonorensis*	Tree	Rutaceae
San Bartolo	Not available	*Carlowrightia arizonica*	Perennial herb	Acanthaceae
San Bartolo	Huirote de corral	*Bignonia unguis-cati*	Vine, annual herb	Bignoniaceae
Santiago	Lomboy blanco	*Jatropha cinerea*	Bush	Euphorbiaceae
Santiago	Palo de arco	*Tecoma stands*	Tree	Bignoniaceae
Santiago	Chicura	*Ambrosia ambrosioides*	Bush	Compositae
Santiago	Vainoro	*Celtis reticulata*	Tree	Ulmaceae
Santiago	Cardón barbón	*Pachycereus pecten-aboriginum*	Cactus tree	Cactaceae
Santiago	Palo chino	*Acacia peninsularis*	Tree	Fabaceae
Santiago	Bledo	*Celosia floribunda*	Tree	Amaranthaceae
Santiago	Guayparin	*Diospyros californica*	Tree	Ebenaceae
Santiago	Hierba del cuervo	*Schaeferia shrevei*	Tree	Celastraceae
Santiago	Mauto	*Lysiloma divaricata*	Tree	Fabaceae
Santiago	Aretito, hierba del alacrán	*Plumbago scandens*	Perennial herb	Plumbaginaceae
Santiago	Cacachila	*Karswinskia humboldtiana*	Tree	Rhamnaceae
Santiago	Crotón	*Croton boregensis*	Shrub	Euphorbiaceae
Santiago	Lomboy rojo	*Jatropha vernicosa*	Bush	Euphorbiaceae
Santiago	Sida	*Sida glutinosa*	Annual herb	Malvaceae
Santiago	Ayenia	*Ayenia glabra*	Annual herb	Malvaceae
Santiago	Caribe	*Cnidosculus angustidens*	Annual herb	Euphorbiaceae
Santiago	Malva colorada, malva rosa	*Melochia tomentosa*	Bush	Sterculiaceae
Santiago	Not available	*Aphanosperma sinaloensis*	Annual herb	Acanthaceae
Santiago	Rama parda	*Ruelia peninsularis*	Shrub	Acanthaceae
Santiago	Not available	*Cissus trifoliata*	Herbaceous vine	Vitaceae
Santiago	Papache	*Randia armata*	Shrub	Rubiaceae
Santiago	Nopal	*Opuntia tapona*	Cactus bush	Cactaceae
Santiago	Choya	*Opuntia cholla*	Cactus bush	Cactaceae
Santiago	Celosa	*Mimosa xantii*	Shrub	Fabaceae
Santiago	Colorin or chilicote	*Erythrina flabelliformis*	Tree	Fabaceae

### Morphometric traits measured in plants (*in-situ*)

#### Plant height, coverage, stems diameter and height of the beginning of canopy

Significant differences between populations were observed in plant height (Table 2). The plants of San Bartolo showed higher height, while lower were showed by plants of Santiago (Table 3). The ANOVA showed no significant differences (Table 2) of plant coverage between populations. Significant differences between populations were observed for main stem diameter (Table 2). Higher values of main stem diameter were found in plants collected in Santiago, followed by San Bartolo plants and the lower values where in plants from Los Gatos (Table 3). The ANOVA showed significant differences between populations for height of the beginning of canopy (Table 2). The plants from San Bartolo showed higher values of this variable respect the plants from Los Gatos and Santiago (Table 3).

**Table 2:** **ANOVA (mean squares) of plant, fruits characteristics and mineral content in tissues (roots, stems and leaves) of wild** ***Capsicum*** **ecotypes collected in three populations near two biosphere reserves in Mexico**

		**Plant**				**Leaf**					**Dry weight**		
Source	d.f.	Height	Coverage	Main stem diameter	Beginning of canopy	Area		Length	Average width	Maximum width	Leaves	Stems	Roots
Populations	2	0.782*	3.81 ns	356.2**	728.46**	194418.39**		0.49 ns	0.41**	1.09**	468.83 ns	129237.91**	54.22 ns
Error	12	0.151	1.11	18.9	118.00	31321.11		0.24	0.04	0.10	349.89	29358.98	357.51
CV (%)		32.40	99.93	34.07	62.91	24.88		10.43	15.32	12.32	78.97	122.37	62.47
		Fruits from collected plants				Four hundred fruits from not collected plants							
	d.f.	Number	Average FW	Average DW	Peduncle length	Length	Width	Seeds per fruit	DW 100 fruits	Seeds DW 100 fruits	Pulp DW 100 fruits	1000 seeds DW	Pulp/seeds ratio
Populations	2	14.08 ns	0.0009 ns	0.0009**	0.05 ns	3.59**	0.19 ns	2.04 ns	0.69**	0.02 ns	0.81**	0.33**	0.13**
Error	9	10.38	0.002	0.0001	0.06	0.23	0.08	3.90	0.06	0.01	0.03	0.05	0.004
CV (%)		39.46	23.20	19.04	10.28	6.27	4.05	12.33	3.77	5.08	3.93	5.54	5.73
								Roots					
	d.f.	Ca	Mg	K		Na		Fe	Mn	Cu	Zn		P
Populations	2	21.60**	1.15**	49.24*		0.10**		1.37 ns	0.001**	0.00002 ns	0.0001**		0.05 ns
Error	12	2.53	0.19	9.53		0.005		0.66	0.0001	0.00001	0.000006		0.03
CV (%)		15.66	23.43	18.24		22.32		77.66	25.30	9.13	5.14		24.47
								Stems					
	d.f.	Ca	Mg	K		Na		Fe	Mn	Cu	Zn		P
Populations	2	6.92 ns	2.89**	239.19**		0.044**		0.001*	0.0002**	0.00002**	0.00006**		0.25 ns
Error	12	3.92	0.36	18.43		0.009		0.0003	0.00003	0.000004	0.000005		0.11
CV (%)		20.61	17.95	12.30		36.97		45.62	20.15	6.23	4.01		17.44
								Leaves					
	d.f.	Ca	Mg	K		Na		Fe	Mn	Cu	Zn		P
Populations	2	67.84**	0.29 ns	224.40**		0.054**		0.00006 ns	0.0002**	0.00005**	0.69 ns		
Error	12	6.28	0.99	24.66		0.71		0.004	0.0001	0.00001	0.000002		0.42
CV (%)		16.59	11.00	7.29		51.96		58.57	24.55	10.85	2.06		25.14

FW = fresh weight. DW = dry weight. d.f. = degree freedom. *Significant probability level *p* ≤ 0.05; **Significant probability level *p* ≤ 0.01. ns = not significant. CV = coefficient of variation.

**Table 3:** **Means of plant, fruits characteristics and mineral content (g kg** ^**−1**^ **dry-weight) in tissues (roots, stems and leaves) of wild** ***Capsicum*** **ecotypes collected in three populations near two biosphere reserves in Mexico**

	**Plant**				**Leaf**					**Dry weight (g)**			
Populations	Height (m)	Coverage (m^2^)	Beginning of canopy (cm)	Stems diameter (mm)	Area (cm^2^)		Length (cm)	Average width (cm)	Maximum width (cm)	Leaves	Stems	Roots	
San Bartolo	1.57 a	2.03 a	31.20 a	12.23 ab	489.25 b		4.36 a	1.08 b	2.09 b	34.29 a	324.75 a	29.32 a	
Los Gatos	1.23 ab	0.77 a	10.60 b	8.61 b	866.45 a		4.91 a	1.63 a	3.03 a	21.45 a	31.80 b	27.54 a	
Santiago	0.78 b	0.35 a	10.00 b	17.58 a	777.55 ab		4.89 a	1.49 a	2.64 a	15.31 a	63.47 ab	33.92 a	
	Fruits from collected plants*				Four hundred fruits from not collected plants**								
	Number	Average FW (g)	Average DW (g)	Peduncle length (cm)	Length (mm)	Width (mm)		Seeds per fruit	DW 100 fruits (g)	Seeds DW 100 fruits (g)	Pulp DW 100 fruits (g)	1000 seeds DW (g)	Pulp/seeds ratio
San Bartolo	8.25 a	0.21 a	0.047 b	2.47 a	7.69 a	7.47 a		16.60 a	7.35 a	3.30 a	4.05 a	4.39 a	1.22 a
Los Gatos	10.0 a	0.22 a	0.078 a	2.52 a	6.67 b	7.04 a		16.23 a	6.53 b	3.38 a	3.15 c	4.07 ab	0.93 b
Santiago	6.25 a	0.19 a	0.061 ab	2.69 a	8.56 a	7.32 a		15.22 a	6.82 b	3.22 a	3.60 b	3.82 b	1.25 a
								Roots					
	Ca	Mg	K		Na			Fe	Mn	Cu	Zn		P
San Bartolo	10.81 a	1.53 b	14.58 b		0.25 b			0.67 b	0.45 ab	0.035 a	0.048 b		0.69 a
Los Gatos	7.83 b	2.41 a	15.68 ab		0.24 b			1.65 a	0.06 a	0.033 a	0.045 b		0.67 a
Santiago	11.83 a	1.62 b	20.48 a		0.49 a			0.83 a	0.37 b	0.037 a	0.054 a		0.87 a
								Stems					
	Ca	Mg	K		Na			Fe	Mn	Cu	Zn		P
San Bartolo	9.24 a	2.90 b	30.98 b		0.20 1b			0.022 b	0.028 ab	0.029 ab	0.058 a		1.65 a
Los Gatos	8.73 a	4.24 a	30.76 b		0.19 b			0.041 ab	0.025 b	0.028 b	0.054 b		1.95 a
Santiago	10.98 a	2.93 b	42.85 a		0.36 a			0.058 a	0.038 a	0.032 a	0.061 a		2.10 a
								Leaves					
	Ca	Mg	K		Na			Fe	Mn	Cu	Zn		P
San Bartolo	14.80 ab	8.77 a	71.14 a		0.77 1			0.06 b	0.055 a	0.036 b	0.064 b		2.97 a
Los Gatos	11.57 b	9.09 a	60.37 b		0.31 b			0.05 b	0.053 a	0.032 b	0.061 c		2.22 a
Santiago	18.92 a	9.25 a	72.66 a		0.45 ab			0.23 a	0.048 a	0.045 a	0.067 a		2.60 a

FW = fresh weight. DW = dry weight. *Each value represents the average of 3 or 10 data set. **Each value represents the average of 100 data set. Means followed by the same letter in each column are not significantly different (Tukey HSD; *p* = 0.05). For mineral content, each value represents the average of five data.

### Growth type

In Los Gatos, 100% of the total plants identified in the population had erect growth (shrub type). In San Bartolo, 73% of the total plants identified had erect growth, while the rest (27%) had climbing growth. In Santiago, 90% of the total plants identified had climbing growth (vine type) while 10% had erect growth.

### Morphometric traits measured in collected plants and fruits (laboratory)

#### Leaf area, leaf length, average and maximum width of leaf

Significant differences between populations were observed for leaf area (Table 2). The higher values of leaf area were in plants from Los Gatos > Santiago > San Bartolo (Table 3). In leaf length, not significant differences between populations were observed. Significant differences between populations were observed in leaf average width (Table 2). High leaf average width was showed in plants collected in Los Gatos followed by plants from Santiago (Table 3). Significant differences between populations were observed for leaf maximum width (Table 2). The higher values of this variable were showed in leaves collected in plants from Los Gatos and Santiago (Table 3).

#### Leaves, roots and stems dry weight

From these variables, leaves and roots dry weights not showed significant differences between populations and only stems dry weight showed significant differences (Table 2) with higher values the plants collected in San Bartolo followed by Santiago (Table 3).

#### Number of fruits per plant, peduncle length and fruit average fresh and dry weights

From these variables, number of fruits per plant, peduncle length and fruit average fresh weight not showed significant differences between populations but only fruit average dry weight showed significant differences (Table 2) with higher values the fruits collected in Los Gatos plants, followed by Santiago (Table 3).

#### Number of seeds per fruit, fruit length and width

Only fruit length showed significant differences between populations (Table 2) with higher length the fruits collected in Santiago, followed by San Bartolo fruits (Table 3).

#### 100 fruits dry weight, seeds and pulp dry weight of 100 fruits, 1000 seeds dry weight and pulp/seeds ratio

One hundred fruits in terms of dry weight showed significant differences between populations (Table 2) with higher values the fruits collected in San Bartolo (Table 3). One hundred seeds dry weight not showed significant differences between populations (Table 2). The variables 100 fruits pulp dry weight, 1000 seeds dry weight and pulp/seeds ratio showed significant differences between populations (Table 2). The fruits collected in San Bartolo showed higher values of 100 fruits pulp dry weight and 1000 seeds dry weight, while the fruits collected in Santiago showed the higher pulp/seeds ratio (Table 3).

#### Mineral content of roots, stems and leaves

The ANOVA of mineral content in roots showed significant differences between populations for Ca, Mg, K, Na, Mn and Zn but not for Fe, Cu and P (Table 2). Calcium, K, Na and Zn was higher in roots of plants collected in Santiago, while the roots of plants from Los Gatos showed higher values of Mg and Mn (Table 3). Significant differences between populations had differences for Mg, K, Na, Fe, Mn, Cu and Zn content in stems and only Ca and P did not show differences (Table 2). The stems of plants collected in Santiago had higher values of K, Na, Fe, Mn, Cu and Zn and only the stems of plants collected in Los Gatos showed higher values of Mg (Table 3). The ANOVA of mineral content in leaves showed significant differences between populations for Ca, K, Na, Fe, Cu and Zn, while Mg, Mn and P did not show significant differences (Table 2). The leaves from plants collected in Santiago had higher values of Ca, K, Fe, Cu and Zn, while the leaves of plants from San Bartolo had higher values of Na (Table 3).

### Relationship of environmental conditions and morphometric traits

Solar radiation of Santiago showed significant correlation (*r* = −0.89, *p* = 0.04) with root dry weight, decreasing as radiation increased. Evapotranspiration was correlated significantly with main stem dry weight in plants collected in Santiago (*r* = −0.87, *p* = 0.05), showing a decresing trend as evapotranspiration increased. The minimum temperature was correlated significantly with leaf average width in Los Gatos (*r* = 0.88, *p* = 0.04) showing an increasing trend as minimum temperature increased. In Santiago, the beginning of canopy decreased as precipitation increased; however, the correlation coefficient was not significant. Also leaf length showed increased as relative humidity increased though the correlation was not significant. In Los Gatos, the maximum leaf width decreased as evapotranspiration increased; however, the correlation was non-significant. Similarly, leaf area showed a trend to increase as minimum temperature increased; however, this correlation was not significant .

## Discussion

The results of MANOVA confirms that there are morphological differences between the three sample populations of wild *Capsicum* plants at the sites studied of Los Gatos, San Bartolo and Santiago in the southern part of Peninsula of Baja California in some of the measured variables. This result strengthens the likelihood that the differences observed in the univariate analysis (ANOVA) performed on the variables, are real differences and not false positives or differences that occur simply by randomized chance [31].

The wild *Capsicum* plants collected in the three populations, showed two types of growth (erect or climbing) in agreement with Vázquez-Dávila [9], and Medina-Martínez *et al*. [32]. Villalón-Mendoza *et al*. [34] reported that some of the species which are associated with wild *Capsicum* plants are nurse plants such as *Helietta parvifolia, Diospyros palmeri, Acacia rigidula, Cordia boissieri, Leucophyllum texanum, Pithecellobium pallens*. They described that the main vegetation types associated with the *C. annuum* ecotypes in northeastern Mexico were thorny shrubs, followed by not thorny shrubs, forests of *Prosopis*, forest of oak-pine and medium size plants that are not thorny shrubs. Lack of abundant rains does not allow for growth of many vegetation types. This was demonstrated in the present study because the sample population with the least abundant variety of plants associated with wild *Capsicum* plants was in Los Gatos with the lowest precipitation, followed by San Bartolo with higher precipitation and Santiago with the highest.

In southern Arizona, U.S.A., where the vegetation is predominantly semi-desert grassland and mesquite woodland [35], Tewksbury *et al*. [36] found a greater association of wild plants of *C. annum* var. *aviculare* [Dierbach] D’arcy and Eshbaugh with seven species. These included *Celtis pallida* Torr., *Condalia globosa* Johnst., *Lycium andersonii* Gray, *Zizyphus obtusifolia* Hook, *Dodonea viscosa* Jacq., *Mimosa biuncifera* Benth., and *Prosopis velutina* Woot. They found that 78% of the plants were established under the canopies of fleshy-fruited shrub and tree species, while notably 58% of the *Capsicum* plants were found under just two species, desert hackberry (*Celtis pallida* Torr.) and netleaf hackberry (*Celtis reticulata* Torr.). A similar relationship has been documented for subtropical thorn scrubs in central Sonora, México, where wild *Capsicum* was 10 times more abundant under fleshy-fruited shrub [37]. In addition, Tewksbury *et al*. [36] also reported that wild *Capsicum* was not found in direct sunlight. Our study is in agreement with these authors, the distribution of *Capsicum* was determined by the micro environmental differences by different nurse-plants species or by nonrandom dispersal by *Capsicum* consumers. Specifically, our study showed that plants of wild *C. annuum* ecotypes in the populations were found to be associated to shrub or tree species, such as was reported by Laborde and Pozo [38] where they indicated that chili piquín was found under 1300 m.a.s.l., regularly in sites in association with shrubs plants where the environmental conditions such as humidity and luminosity are appropriate.

Leaf length of *Capsicum* plants from Santiago increased as relative humidity increased suggests that high morphometric variables are not necessarily related to environmental conditions, since leaf length values were higher in those plants from Los Gatos, where relative humidity was the lowest compared to the other sites. San Bartolo had high while Santiago intermediate values of relative humidity. In addition, root dry weight of plants collected in Santiago decreased as solar radiation increased. However, Santiago showed intermediate values of solar radiation compared to Los Gatos (the highest values) and San Bartolo (the lowest values). Our study showed that wild *Capsicum* plants were found under 700 m.a.s.l. which coincide with the reported by Laborde and Pozo [38] and Villalón-Mendoza *et al*. [34] where they stated that wild *Capsicum* species is commonly found with thorn scrubs at altitude limits at 600–800 m.a.s.l.

Medina-Martínez *et al*. [11] in a study of wild *C. annuum* in the northeast Mexico found that wild *Capsicum* can growth under high temperatures during summer season (up 40°C) with partial shade and were associated mainly with leguminous species. In a later study by also Medina-Martínez *et al*. [32] wild chili pepper populations were commonly found at intermountain and piedmont sites. They found that they grow mainly in vertisol and rendzins soil types, although less frequently in the later. The plants were found to be perennial with growth increasing with spring rains that produce fruits in summer and autumn to be commercialized by families in rural communities.

In the present study all wild *Capsicum* plants were found under shrubs and trees. The temperatures (20–30°C) of the autumn season (September, October and November) in the zone were conducive to wild *Capsicum* plants because flowering and seedling development improved and fruits production increased. The results are in agreement with the evidence showed by Heiser and Pickersgill [39] where they described that wild chilies identified as *Capsicum annuum* var. *glabriusculum*, commonly known as “chiltepines” are widely distributed in Mexico, especially under tree species of tropical deciduous forest, also it is possible found around field crops and to roadsides. Medina-Martínez *et al*. [32] stated that *C. annuum* var. *aviculare* grew favorably under clay-loam texture soils with pH of 7.5 and electrical conductivities between 0.5-1.0, with high organic matter content (3.5% on average) containing elements such as nitrogen, phosphorus and potassium. Our study showed that wild *Capsicum* plants were found in a range of temperature among 22 to 23° C, with maximum of 33°C, minimum of 13°C and average of 22.5° C which coincide with those reported by Medina-Martínez *et al*. [33].

*Capsicum* species occur in a wide range of different habitats with an average day temperature between 7 and 29°C, an annual precipitation between 300 and 4600 mmand a soil pH between 4.3 and 8.7 [40]. In general, *Capsicum* species are cold sensitive and grow best in well-drained, sandy or silt-loam soil [40].

In the present study, 70% of plants had significant morphometric differences between populations, while in fruits, 50% showed significant differences. It is important to note, that other studies of wild *Capsicum* have reported a high variability of morphometric traits such as main stem and foliage characteristics where the foliar covering or diameter was found to have a range of 0.60-1.05 m, in the plant height of 30–98 cm, in the leaves length of 1.9-4.2 cm and in leaf width of 1.1-2.3 cm and about the fruits production, high variability was appreciated in the precocity degree, fruit length and width and yield of fruits per plant [41]. The fruit length range of 1.1-2.5 cm and the fruit width was 0.5 at 1.0 cm [41]. In the same sense, Medina-Martínez *et al*. [32] reported a high variability between morphometric traits in chili piquín (*C. annunm* var*. aviculare*) with an average of 2.8 cm in leaf width, plant height of 2.0 m, length of petioles of 5–20 mm, fruit peduncle length of 1–2 cm and diameter of 0.5 mm, the fruit is a berry from 8–10 mm of length and 5–8 mm of width, with yellowish brown seeds of 2.5 mm of length. Because the fruit or pod, technically a berry, is the commodity of the pepper plant, fruit morphology flavor and pungency are the characteristics of most economic importance within the genus. A tremendous wealth of genetic variation is known with respect to fruit traits such as size, shape, color, and flavor, resulting in more than 50 commercially recognized pod types. The major pod types are described by Bosland [42], Andrews [43] and by Paran et al. [44].

Other studies in wild populations of *C. annuum* from northwest Mexico have found a high variation in morphometric traits such as fruit length (range 0.30-0.98 cm) and seed number (range 1–34) in same populations [16]. In other latitudes of the world, similar results have been reported. Shrilekha Misra *et al*. [45] reported that in 38 accessions of *C. annuum* collected from diverse locations in India, divergence of pooled characters ranged from 41–111 cm plant height, 6.62-45.39 cm^2^ leaf surface area, 1.45-9.96 cm fruit length, 0.65-1.84 cm fruit diameter, 2.64-27.40 cm^2^ fruit surface area, 0.36-4.447 mg fruit fresh weight and 0.14-0.96 mg fruit dry weight. Hernández-Verdugo *et al*. [46] reported high variability in 11 morphometric traits, except for main stem diameter which showed values between 1.1-1.8 cm in seven wild *Capsicum* populations in different habitats in Sinaloa, México. The measured morphometric traits were plant height (95–181 cm), plant width (68–175 cm), main stem length (21–61 cm), leaf width (1.4-3.3 cm), leaf length (3.5-5.6 cm), pedicel length (2.3-2.8 cm), fruit width (5.5-7.7 mm), fruit length (5.6-7.6 mm), number of seeds per fruit (11–17) and seed weight (1.9-2.7 mg) [46]. Some traits measured in the present study are between the range values with those found by Hernández-Verdugo *et al.* [46].

The results of our study show high morphometric variability between the populations of wild *C. annuum* in three sites near two reserve biospheres in Baja California Sur, Mexico. The phenotypic diversity and undoubtedly the genetic diversity of wild *Capsicum* in each of these populations are affected by geography, climate, ecology and human intervention. The trend of stem dry weight to decrease as evapotranspiration increased in those plants of Santiago suggests that evapotranspiration is an important climatic variable in the growth, production and yield of wild *Capsicum*. Higher evapotranspiration was found for plants measured in Los Gatos, followed by Santiago and San Bartolo. The main stem dry weight was higher in San Bartolo plants followed by Santiago which showed the lowest values in those plants collected in Los Gatos but this sample population showed the higher values of evapotranspiration. Also, the maximum leaf width showed a trend to decrease as evapotranspiration increase in those plants collected in Los Gatos. According to Brown [47] an improved understanding of climate effects on the current structure of genetic diversity and morphometric variation within the species is important for efficient germplasm conservation and use.

In the present study, the significant differences found in population site morphometrics could be related to environmental condition(s) where the wild *Capsicum* populations are found. For example, the plants collected in two populations (San Bartolo and Santiago) near La Laguna reserve biosphere showed higher values in the majority of morphometric traits in both plants and fruits compared to Los Gatos probably because these populations are close to the Tropic of Cancer where the precipitation is higher. The Los Gatos population is close to the El Vizcaino reserve biosphere. Nevertheless, in spite of the lower amount of precipitation the wild plants collected in Los Gatos showed more vigor because length, area, average width and maximum leaf width were higher respect with respect to San Bartolo and Santiago plants. Leaf average width in those plants collected in Los Gatos increased as minimum temperature increased. Similarly, the leaf area showed a trend to increase as minimum temperature increase in Los Gatos. The results of both variables show that the range of temperature for better growth of this species is when temperature is higher than 13° C. Also, these differences could be an evidence that ecotype from Los Gatos differ genetically from the ecotypes collected in San Bartolo and Santiago; however, more studies related to genetic, physiology, botanical, and others topics are required. Evidently the differences in environmental conditions such as temperature, nutrient availability and altitude have an influence on plant growth [48]. In the present study, the micro-environmental conditions in the three different sample populations, such as temperature, photoperiod, light quality and nutrient availability suggest that they may be sufficiently distinct to have caused the observed differences in morphometric traits in both plants and fruits, also the mineral content of roots, stems and leaves of wild *Capsicum* plants may also pay a role. The mineral content in roots, stems and leaves is an important variable that influences the plant response under different environmental conditions. Our study showed that plants from Santiago had the higher values of Ca, K, Cu, Zn and P in roots, stems and leaves, higher values of Na in roots and stems, Fe in stems and leaves and Mg in leaves. Although plants from Santiago showed good nutrition condition, they did not necessarily have higher values of morphometric traits in both plants and fruits; however, these plants showed higher values of main stem diameter and root dry weight, also in some morphometric traits in fruits such as peduncle length, fruit length and pulp/seeds ratio. Recently, research regarding the identification of hot pepper cultivars containing low Cadmium levels after growing on contaminated soil [49] and protective role of Selenium on pepper exposed to Cadmium stress during reproductive stage [50] have been reported. Cadmium and other non-essential and highly toxic elements to plants, can pose a human health risk throughout the food chain. Future work will be carried out to determine whether these cultivars are low or high Cd accumulation plants. This is essential if this crop is developed in the future as a commercial product for human consumption, since low Cd cultivars are preferred for human health reasons.

## Conclusions

This is the first study evaluating the ecology and morphometric traits of both plants and fruits of wild *C. annuum* in Baja California Sur, Mexico. The results provide useful information regarding morphometric variation between wild *Capsicum* populations. This could prove valuable to future decision processes involved in the management and preservation of germplasm and genetic resources. The wild relatives of cultivated *C. annuum* are a valuable genetic resource that needs to be conserved. Probably, the populations of wild relatives of chili here in the Peninsula of Baja Calironia due to its geographic isolation maintain high levels of genetic, ecological variability, and are potentially useful genes for agriculture. Future studies are nneded that will evaluate *C. annuum* in the study area to investigate genetic differentiation for upcoming plant breeding efforts with *Capsicum*. There remain some areas of interest in the Peninsula that should be visited in the future, for example, Sierra of La Giganta in front of Loreto City, Sierra of Mulegé in front of Mulegé town, and other sites of the Region of the Cape in the southern part of the Baja California Peninsula. These areas should be a target for future data collection and investigation, including ethnobotanical studies, providing a seed sample bank that will be publicly available for research in plant improvement and for subsequent use in an inquiry into the domestication of *C. annuum*.

## Methods

### Ethics statement

The research conducted herein did not involve measurements with humans or animals. The study site is not considered a protected area. No protected or endangered or species were used in the course of carrying out this study, however, some special permissions need to be get at the Procuraduría Federal de Protección al Ambiente (PROFEPA) at La Paz, Baja California Sur, México. *Capsicum annuum* used in the present study is not considered an endangered species and their use therefore had negligible effects on broader ecosystem functioning.

### Sampling populations

**Figure 1:**
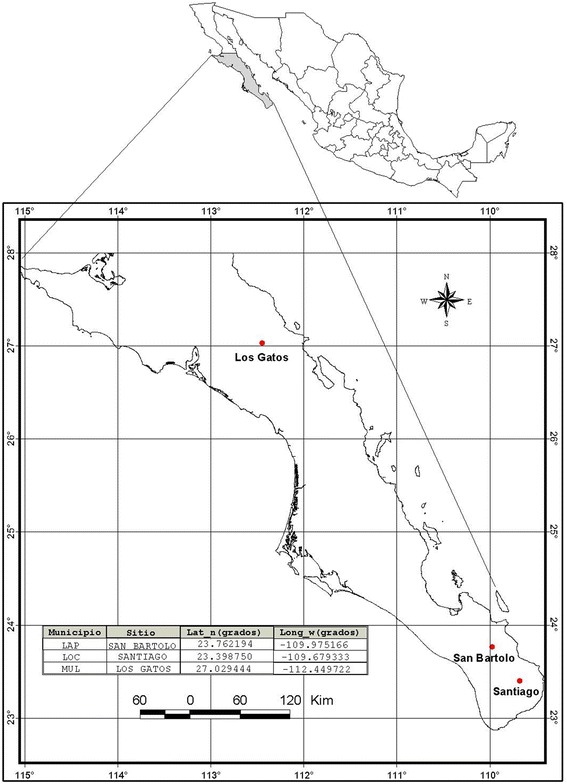
Localization of wild *Capsicum* ecotypes collected in three populations near two biosphere reserves in Mexico.

**Figure 2:**
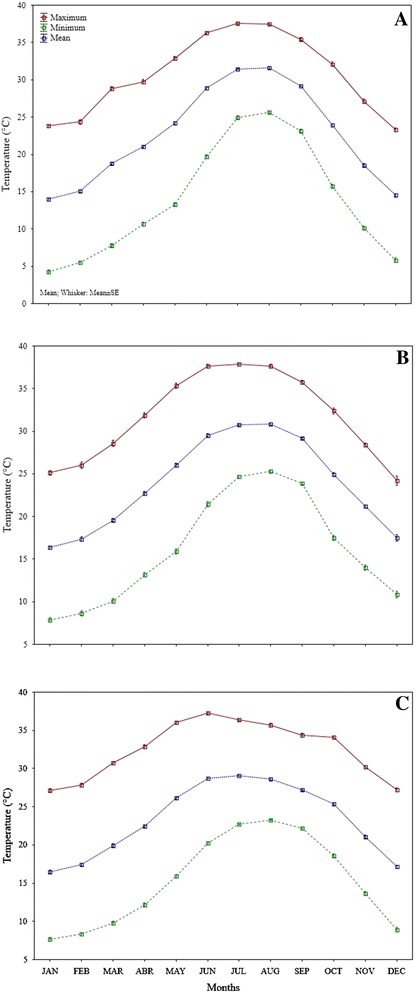
Maximum, minimum and mean temperature of three populations, Los Gatos **(A)**, San Bartolo **(B)** and Santiago **(C)** of wild *Capsicum* ecotypes collected near two biosphere reserves in Mexico.

**Figure 3:**
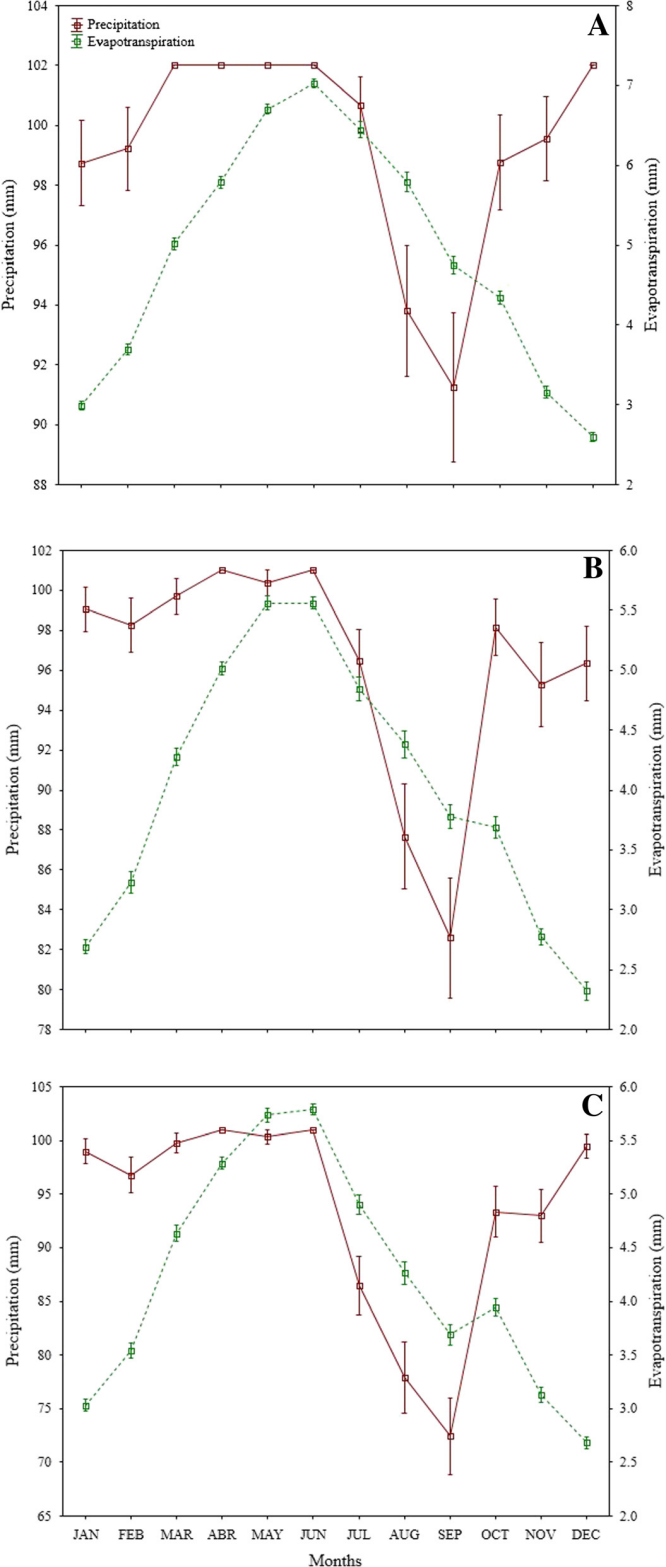
Precipitation and evapotranspiration of three populations, Los Gatos **(A)**, San Bartolo **(B)** and Santiago **(C)** of wild *Capsicum* ecotypes collected near two biosphere reserves in Mexico.

**Figure 4:**
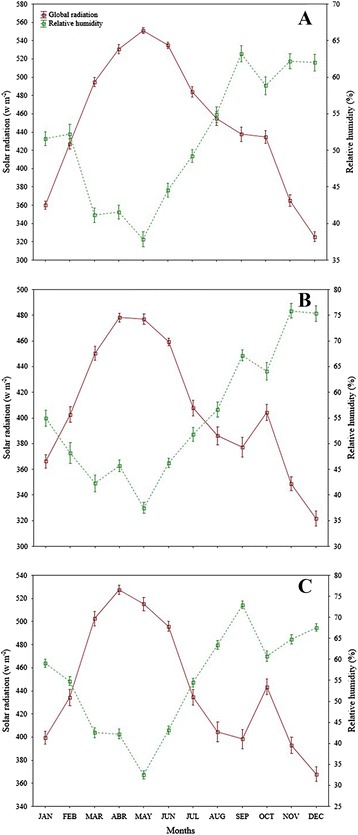
Solar radiation and relative humidity of three populations, Los Gatos **(A)**, San Bartolo **(B)** and Santiago **(C)** of wild *Capsicum* ecotypes collected near two biosphere reserves in Mexico.

Three populations (Figure 1) were located in three sites along Baja California Sur (B.C.S.), Mexico to identify wild *C. annuum* ecotypes. The three sample wild populations were selected based on information provided by local inhabitants in each municipality of Baja California Sur. This data of wild *Capsicum* plants was assessed in extensive field trips and respective interviews with communities and farmers located in wild areas, i.e. in Mulegé municipality, the population of Santa Lucia mountain with more abundance in wild *Capsicum* plants is the area called Los Gatos (The Cats) and surroundings. The sample populations were positioned geographically using a global positioning system (Garmin GPS Map 60Cx). One population was situated in the first site, which was in the municipality of Mulegé (Los Gatos Ranch) near the limit area of biosphere reserve El Vizcaino, B.C.S., México. The second population was located in a second site in the municipality of La Paz (San Bartolo town) and the third population of the third site was located in the municipality of Los Cabos (Santiago town) both near the area limit of the biosphere reserve La Laguna, B.C.S., México. Los Gatos is located in a semiarid zone of Baja California Sur, northwest of Mexico (27°01′46″ N, 112°26′59″ W), 680 meter above sea level (masl). Los Gatos is a wild *Capsicum* population surrounded by some cattle ranches, located in a small range just behind Santa Rosalía, B.C.S., at Santa Lucia Mountain, which joins the Sierra of Guadalupe to the south. This wild *Capsicum* population is located around the limits of El Vizcaino biosphere reserve, close to the highest hill called La Bandera. Below the Pacific slopes of the mid-peninsular range, the Central Desert stretches from 30°N to 26°N and encompasses the Vizcaino Desert and, to the south of the Madgalena Plain. The soils of this population are shallow, of recent formation and high rate of erosion, characterized as lithosol soils, with low organic matter, have no structure to be composed of unconsolidated material with high sand content. Are set on hills and mountain areas, where the type of vegetation is found of sarcocaule scrub. Are coarse textured and are associated with eutric regosols. San Bartolo is located in a subtropical zone of Baja California Sur, northwest of Mexico (23°45′43.9″ N, 109°58′30.6″ W), 526 masl. The wild *Capsicum* population of San Bartolo is located around the limits of La Laguna biosphere reserve, near Sierra of La Laguna lies below La Paz in the Cape Region. The range is called La Laguna after a mountain meadow that, according to natives, was once a lake. This wild *Capsicum* population is close to Arroyo (Dry River) of San Bartolo that it is large. This population is located in the east face of La Laguna Mountain, with high precipitation, with deep canyons and luxuriant growth found on many of these gradual eastern slopes. The soils of this population are predominantly eutric cambisol, a weakly developed mineral soils in unconsolidated materials, soil management affects moisture-holding capacity, the highest moisture contents is found in undisturbed soils, which are related to low organic matter contents, medium to low porosity and low values of structural stability. Santiago is located in a subtropical zone of Baja California Sur, northwest of Mexico (23°23′55.5″ N, 109°40′45.6″ W), 226 masl. The wild *Capsicum* population of San Santiago is located around the limits of La Laguna biosphere reserve, near Sierra de La Laguna lies below La Paz in the Cape Region. The range is called La Laguna after a mountain meadow that, according to natives, was once a lake. This wild *Capsicum* population of Santiago is close to Arroyo (Dry River) of San Bernardo and Arroyo of San Dionisio, both are large. This population is located in the southeast face of La Laguna Mountain, is steep, with deep canyons and luxuriant growth found on many of the more gradual eastern slopes. The soils of this population are dominated by eutric cambisol that with natural vegetation had the highest moisture-holding capacity, the highest rates of infiltration are found for natural vegetation soils, structural profile and porous system are more stable in unchanged soils. Figures 2, 3 and 4 shows the environmental conditions such maximum, minimum and average temperature (°C), precipitation (mm), evapotranspiration (mm), solar radiation (w m^−2^) and relative humidity (%) of the three sample populations in a range of 73 years from 1939 to 2013 along January to December (monthly average). The meteorological observations were obtained during the study from an automated weather stations located at the study areas which are property of the National Institute of Forestry, Agricultural and Livestock Research (INIFAP) and from the National Weather Service (SMN) both institutions of the Secretary of Agriculture, Livestock, Rural Development, Fisheries and Food (SAGARPA) with coverture in all regions of Mexico.

### Vegetation associated to wild *Capsicum* (*in-situ*)

In each sample population, two rectangles of 50 × 20 m (1000 m^2^) were traced and each *Capsicum* plant were counted and identified in each rectangle. One square of 4 × 4 m (16 m^2^) was traced around each *Capsicum* plant found and the vegetation associated was identified the family, common and scientific names.

### Morphometric traits measured in plants (*in-situ*)

In each sample population, five wild *Capsicum* plants were selected completely randomized and the height (cm), plant coverage (m^2^), main stem diameter (mm), as well as the height of the beginning of canopy (cm) were measured. We collected only five plants of each sample population since the Procuraduría Federal of Protección to the Ambiente (PROFEPA) authorized only the collection of a limited number of wild *Capsicum* plants and fruits. This species is perennial, however annual growth change yearly, thus this first study will be important for providing the baseline for future growth studies of this plant species. Plant coverage, plant height and height of beginning of canopy were measured using a metric tape of 5 m and main stem diameter was measured using a digital caliper (General No 143, General Tools®, Manufacturing Co., Inc., New York, USA) at a plant height of 0.20, 0.40 and 0.60 m and the result was averaged. The growth types of all *Capsicum* plants found in each sample population were recorded. The growth type was identified as two types, as erect (shrub type) or climbing (vine type).

### Plants and fruits collection

Previously to realizing the collection, a specific permission needs to be granted by PROFEPA in La Paz, Mexico in order to collect wild *Capsicum* plants and fruits. These plants at present not considered endangered or protected species. However, for future plants and fruits collection of *Capsicum* and other species in the sample populations near both biosphere reserves will be sampled after attaining appropriate permissions contacting to Mr. Leonel Valerio Castro Santana, Federal Officer of PROFEPA in Baja California Sur. In each sample population, the five plants of wild *Capsicum* selected were collected and completely randomized (including roots). These plants were used for morphometric measurements. Each plant was considered as a replication. The collected plants were introduced in paper bags, labelled, stored in cardboard containers and moved to the laboratory of plant physiology at Centro de Investigaciones Biológicas del Noroeste, S.C. (CIBNOR®) at La Paz, México. Before the collection of each plant, the total fruits per plant were harvested and placed in paper bags, labelled and stored in a cardboard container and moved to the laboratory. At the same time, 400 mature fruits from different plants (without collecting) at each sample population were collected, introduced in paper bags, labelled and moved to the laboratory. Each group of 100 fruits was considered as one replication. We collected 400 mature fruits because PROFEPA authorized only the collection of this quantity of wild *Capsicum* fruits based on the criteria of the normativity for wild vegetation in Mexico considering criteria for conservation and management of resources.

### Morphometric traits measured in collected plants and fruits (laboratory)

In the laboratory, the five wild *Capsicum* plants collected were separated into roots, leaves and stems and the following variables were measured:

#### Leaf area, leaf length, average and maximum width of leaf

Leaf area (cm^2^), leaf length (cm), average (cm) and maximum (cm) width of leaf of each collected plant of each sample population that was collected was measured with a Li-Cor portable leaf area meter (Li-Cor®, modelo-Li-3000A, series Pam 1701, Li-Cor® Lincoln, Nebraska, USA).

#### Leaves, roots and stems dry weights

All leaves, roots and stems dry weights from each plant collected in each sample population were recorded. The leaves, roots and stems were placed in a pre-heated oven (Shel-Lab®, model Fx-5, serie-1000203) at 80°C, until constant weight, in order to obtain leaves (g), roots (g) and stems (g) dry weights which were obtained using a conventional scale (Ohaus®, model CT600-S, USA, series 18939).

In the laboratory, the 400 fruits harvested from each sample population and those fruits collected *in-situ* were separated into peduncle, seeds and fruit pulp and the following variables were measured:

#### Number of fruits per plant, peduncle length and fruit average fresh and dry weights

Each fruit collected from each collected plant were counted and recorded. The peduncle of each fruit was separated from the fruit and the length (cm) was recorded using a digital caliper (General No 143, General Tools®, Manufacturing Co., Inc., New York, USA.). Average fresh weight of fruit (g) was determined using a conventional scale (Ohaus®, model CT600-S, USA, series 18939) and average dry weight of fruit (g) were obtained when each group of fruits from each plant were placed in a pre-heated oven (Shel-Lab®, model Fx-5, serie-1000203) at 80°C, until constant weight.

#### Number of seeds per fruit, fruit length and width

The 400 mature fruits collected from each sample population were used to determine the number of seeds per fruit, length (mm) and width (mm) of fruit which were measured using a digital caliper (General No 143, General Tools®, Manufacturing Co., Inc., New York, USA.).

#### 100 fruits dry weight, seeds and pulp dry weight of 100 fruits, 1000 seeds dry weight and pulp/seeds ratio

The 400 mature fruits collected from each sample population were separated in four groups of 100 fruits and fruits dry weight (g), seeds (g) and pulp (g) dry weight, pulp/seeds ratio and 1000 seeds dry weight (g) were measured. The dry weight were obtained when the fruits or seeds were introduced in a pre-heated oven (Shel-Lab®, model Fx-5, serie-1000203) at 80°C, until constant weight.

#### Mineral content of roots, stems and leaves

The mineral content in roots, stems and leaves is an important variable that influences the plant response under different environmental conditions. All roots, leaves and stems after being separated from the main plant were rinsed by dipping three times for a few seconds in distilled-deionised water before measuring dry weights. Separately roots, leaves and stems dried tissue were finely ground in a blender (Braun® 4–041 Model KSM-2) for mineral analysis. The Na, Ca, Mg, Mn, Fe, Cu, Zn, and K (all in g kg^−1^ dry-weight) content was determined by atomic absorption spectrophotometer (Shimadzu AA–660, Shimadzu®, Kyoto, Japan) after digestion with H_2_SO_4_, HNO_3_, and HClO_4_. Phosphorous (g kg^−1^ dry-weight) was estimated colorimetrically as phosphomolybdate blue complex method at 660 nm from the same extract.

### Statistical analysis

Bartlett’s test was performed on the data to test the homogeneity of variance. Data were analyzed using a fit model using a standard least squares means personality function and univariate and multivariate analysis of variance (ANOVA and MANOVA). All plants and fruits variables were analyzed for one way of classification, being sample population the study factor. The least significant differences were calculated using Tukey’s HSD test (*p* ≤ 0.05) when the analysis of variance was significative. As a wild population, the coefficient of variation for each variable was considered. In all cases, differences among means were considered significant at *p* ≤ 0.05. Single and multiple Pearson’s correlation coefficients (*r*) at 95% confidence limits for independent variables (environmental conditions) and dependent variables measured in plants, fruits and seeds was determined. All analyses were done with Statistica software program v. 10.0 for Windows.

### Availability of supporting data

The authors confirm that all data underlying the findings are fully available without restriction. All the relevant data that is needed to replicate this study and to draw the conclusions for this study is within the paper.
